# ICTV Virus Taxonomy Profile: Mymonaviridae

**DOI:** 10.1099/jgv.0.001301

**Published:** 2019-09-03

**Authors:** Dàohóng Jiāng (姜道宏), María A. Ayllón, Shin-Yi L. Marzano, ICTV Report Consortium

**Affiliations:** 1 The State Key Laboratory of Agricultural Microbiology, College of Plant Science and Technology, Huazhong Agricultural University, Wuhan 430070, PR China; 2 Centro de Biotecnología y Genómica de Plantas (CBGPUPM-INIA), Universidad Politécnica de Madrid (UPM) - Instituto Nacional de Investigación y Tecnología Agraria y Alimentaria (INIA), Campus de Montegancedo-UPM, 28223-Pozuelo de Alarcón (Madrid), Spain; 3 Department of Biology and Microbiology, Department of Agronomy, Horticulture, and Plant Sciences, South Dakota State University, Brookings, South Dakota, USA

**Keywords:** *Mymonaviridae*, mymonavirus, ICTV Report, sclerotimonavirus, taxonomy

## Abstract

Members of the family *Mymonaviridae* produce filamentous, enveloped virions containing a single molecule of linear, negative-sense RNA of ≈10 kb. The family currently includes a single genus, *Sclerotimonavirus*. Mymonaviruses usually infect filamentous fungi, and one virus, Sclerotinia sclerotiorum negative-stranded RNA virus 1, induces hypovirulence in the fungal host. This is a summary of the International Committee on Taxonomy of Viruses (ICTV) Report on the family *Mymonaviridae*, which is available at ictv.global/report/mymonaviridae.

## Virion

**Fig. 1.**
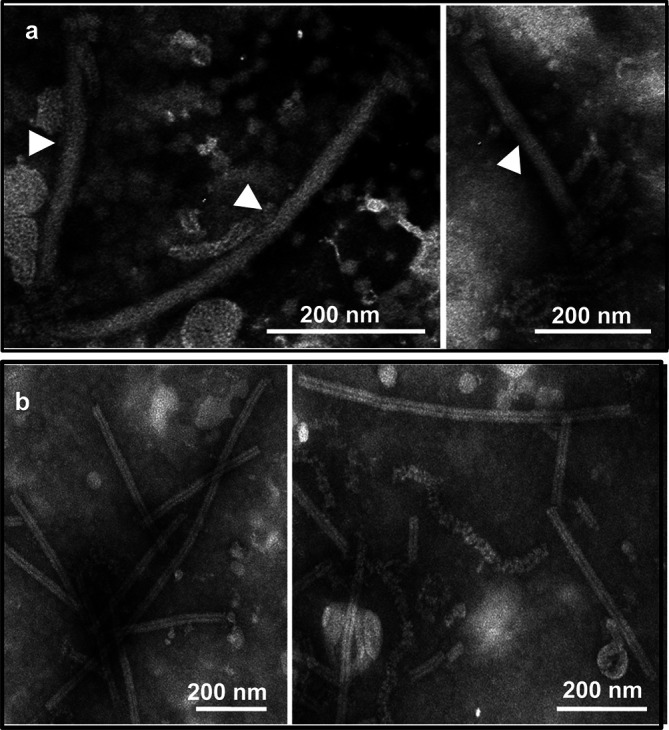
Morphology and structure of virions and nucleoprotein–RNA complexes (RNPs) of Sclerotinia sclerotiorum negative-stranded RNA virus 1. (**a**) Filamentous, possibly enveloped virions (marked by white triangles, left) and ribonucleoprotein (RNP) complexes (right). (**b**) Purified tight (left) or loose (right) coils of RNP complexes.

Virions are filamentous, 25–50 nm in diameter, ≈1000 nm in length and enveloped by a membrane (Table 1). The outer surface of virions does not appear to be covered with spikes. The nucleocapsids released from virions are single, left-handed, helical structures that, when tightly coiled, have a diameter of 20–22 nm and a length of 200–2000 nm. The nucleocapsids consist of aggregated nucleoprotein (NP) monomers (Fig. 1).

**Table 1.** Characteristics of members of the family *Mymonaviridae*

Typical member:	Sclerotinia sclerotiorum negative-stranded RNA virus 1 (KJ186782), species *Sclerotinia sclerotimonavirus*, genus *Sclerotimonavirus*
Virion	Enveloped, filamentous virions 25–50 nm in diameter and ≈1000 nm in length
Genome	Single molecule of linear, negative-sense RNA of ≈10 kb
Replication	Ribonucleoprotein (RNP) complexes containing anti-genomic RNA serve as templates for synthesis of nascent RNP complexes containing genomic RNA
Translation	The viral RNA-directed RNA polymerase binds the encapsidated genome at the leader region and then sequentially transcribes each gene by recognizing start and stop signals flanking viral genes. This produces subgenomic RNAs that serve as mRNAs
Host range	Fungi
Taxonomy	Realm *Riboviria,* phylum *Negarnaviricota*, subphylum *Haploviricotina*, class *Monjiviricetes*, order *Mononegavirales,* family *Mymonaviridae*, genus *Sclerotimonavirus* with >five species assigned

## Genome

**Fig. 2.**
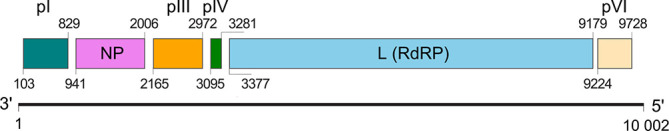
Genome organization and characteristics of Sclerotinia sclerotiorum negative-stranded RNA virus 1. The position of each ORF is indicated above the negative-sense strand. The ORFs putatively encode six proteins: pI, NP (nucleoprotein), pIII, pIV, L (RdRP, RNA-directed RNA polymerase) and pVI.

Virions contain a single molecule of a linear, negative-sense RNA genome of ≈10 kb. The genome lacks a poly(A) tail at the 3′-terminus and is uncapped at the 5′-terminus. The two termini are not complementary in sequence. The mymonavirus genome is predicted to have six major non-overlapping open reading frames (ORFs I–VI). These ORFs are expressed as individual transcription units and are separated by non-coding intergenic regions containing highly conserved gene junction sequences. Mymonaviruses express at least six proteins (Fig. 2). The nucleoprotein encapsidates the mymonavirus genome. The RNA-directed RNA polymerase (RdRP, part of the Large protein - L) mediates viral genome replication and transcription. The functions of the remaining four proteins are unclear.

## Replication

Mymonaviruses are believed to replicate in the fungal cytoplasm, but their replication strategy is not well studied. Ribonucleoprotein (RNP) complexes can be used directly as templates for replication and transcription. Replication usually occurs on RNP complexes and requires L to synthesize full-length positive-sense antigenomes that serve as a template for the synthesis of negative-sense progeny genomes.

## Pathogenicity

Sclerotinia sclerotiorum negative-stranded RNA virus 1, a typical mymonavirus that is isolated from *Sclerotinia scleoriotum*, causes hypovirulence phenotypes, including slow growth and loss of pathogenicity, in its host (rapeseed). Sclerotinia sclerotiorum negative-stranded RNA virus 1 can be transmitted horizontally through hyphal fusion [1].

## Taxonomy

Mymonaviruses form a family in the haploviricotine order *Mononegavirales*. Within this order, mymonaviruses are most closely related to members of the families *Bornaviridae* and *Nyamiviridae*.

Viruses in the genus *Sclerotimonavirus* have been characterized in three phytopathogenic fungi – *Sclerotinia sclerotiorum* [1,3]), *Botrytis cinerea* [4] and *Fusarium graminearum* [5] – and have also been discovered in soybean phyllosphere phytobiomes [6] and an invertebrate [7].

## Resources

Current ICTV Report on the family *Mymonaviridae*: ictv.global/report/mymonaviridae.

## Abbreviations

L: Large protein
NP: Nucleoprotein
RdRP: RNA-directed RNA polymerase
RNP: Ribonucleoprotein
